# A meta-analysis on the role of sonication in the diagnosis of cardiac implantable electronic device-related infections

**DOI:** 10.3389/fmicb.2024.1361626

**Published:** 2024-03-15

**Authors:** Daniela Araújo, João P. Martins, Stephanie Lopes Ferreira, Sandra Mota, Pedro L. Ferreira, Rui Pimenta

**Affiliations:** ^1^Escola Superior de Saúde, Instituto Politécnico do Porto, Rua Dr. António Bernardino de Almeida, Porto, Portugal; ^2^CEAUL– Centro de Estatística e Aplicações, Faculdade de Ciências, Universidade de Lisboa, Campo Grande, Lisbon, Portugal; ^3^CHUdSA – Centro Hospitalar Universitário de Santo António, Porto, Portugal; ^4^REQUIMTE/LAQV, Escola Superior de Saúde, Instituto Politécnico do Porto, Rua Dr. António Bernardino de Almeida, Porto, Portugal; ^5^Faculty of Economics, University of Coimbra, Coimbra, Portugal; ^6^Centre for Health Studies and Research of University of Coimbra, Centre for Innovative Biomedicine and Biotechnology, Avenida Dias da Silva, Coimbra, Portugal

**Keywords:** sonication, implant associated infections, cardiac implantable electronic devices, biofilms, microbiological diagnosis, systematic review, meta-analysis

## Abstract

**Introduction:**

One of the biggest obstacles in diagnosing Implant-Associated Infections is the lack of infection criteria and standardized diagnostic methods. These infections present a wide range of symptoms, and their diagnosis can be hampered by the formation of microbial biofilms on the surface of implants. This study aimed to provide insight into the performance of sonication in the diagnosis of infections associated with Cardiac Implantable Electronic Devices, to help define a consensus on the algorithm for the microbial diagnosis of these infections.

**Methods:**

We carried out a systematic review with meta-analysis. The PRISMA methodology guidelines were followed, and an advanced search was carried out in PubMed and Web of Science, which enabled 8 articles to be included in the review, in which a meta-analysis was also carried out. QUADAS-2 was used to assess the risk of bias and effect measures were calculated to assess publication bias.

**Results:**

The overall sensitivity of the method was 0.823 (95% CI: 0.682–0.910) and the specificity was 0.632 (95% CI: 0.506–0.743).

**Discussion:**

These results suggest that sonication may offer advantages in diagnosing these infections. However, it is essential to approach these findings carefully and take into account the recommendations provided in the EHRA 2019 guidelines. This study highlights the importance of more effective diagnostic approaches for implantable medical device-associated infections to improve the quality of treatment and minimize the risks associated with these challenging medical conditions.

## 1 Introduction

Despite the advances in sterilization and aseptic techniques, Implant-Associated Infections (IAIs) remain a serious problem with significant morbidity and economic burden (Kurtz et al., 2012; Kamath et al., 2015). IAIs can lead to chronic pain, systemic infections, or revision surgeries for implant replacement and removal of infected tissues (Oliva et al., 2021).

The mortality rate of IAIs depends on the type of implant, with intravascular implants posing the highest risk. In the coming years, due to the aging of the population and the rise of underlying comorbidities, it is expected that the incidence of these infections to increase (Darouiche, 2001; VanEpps and Younger, 2016).

The infections associated with Cardiac Implantable Electronic Devices (CIED) are included in IAIs. CIED are medical devices implanted to regulate and/or monitor the heart rhythm. This category includes Pacemakers, Implantable Cardioverter-Defibrillators (ICDs), Cardiac Resynchronization Therapy devices (CRTs), and Implantable Loop Recorders (ILRs) (Haghjoo, 2017).

Typically, CIED consist of an electric pulse generator and one or more leads that connect the generator to the interior of the cardiac cavities (Haghjoo, 2017). Thus, CIED infections can be local, such as localized infection of the generator pocket, or develop into a systemic infection or infective endocarditis (IE) (Blomström-Lundqvist et al., 2020).

Pocket Infection (PI) is defined as a local infection of the generator pocket. A low-grade or indolent infection may include erythema, increased local temperature, and deformations of the pocket. These signs can evolve into skin adhesion to the device and dehiscence of the surgical wound with purulent discharge. PI may be associated with lead infections, systemic infections associated with CIED, and IE. However, these manifestations can also occur without PI association. The diagnosis of these cases is challenging since symptoms are very nonspecific (fever, chills, night sweats) and may only manifest after long periods following CIED implantation (Blomström-Lundqvist et al., 2020).

Currently, there are no standard criteria for diagnosing endocarditis associated with CIED. Modified Duke criteria and the 2015 ESC criteria have been accepted; however, these do not represent standard tools and are not validated for these specific cases. To update the state of the art regarding CIEDIs, the European Society of Cardiology (ESC) created an international consensus document in 2019 (Blomström-Lundqvist et al., 2020). This document reflects the joint effort of several organizations to upgrade the management of these infections. It is a combination of modified Duke criteria and ESC criteria, along with some additional criteria (Blomström-Lundqvist et al., 2020).

It describes the current knowledge about the risk of infections associated with CIED and aims to assist healthcare professionals in decision-making regarding their prevention, diagnosis, and management, providing updated information on the most recent and effective strategies (Blomström-Lundqvist et al., 2020).

The EHRA 2019 guidelines establish an algorithm to reach a definitive or possible diagnosis of Infection Associated with CIED or IE, or its rejection, through the satisfaction of Major and Minor Criteria. The Major criteria include the results of Microbiology and Imaging techniques. Minor criteria include other clinical signs such as fever or predisposing health conditions (Blomström-Lundqvist et al., 2020).

The hypothesis of biofilm formation on the surface of implants may explain the difficulties in diagnosing and treating IAIs, including those associated with CIED (Darouiche, 2001; Drago and De Vecchi, 2017). The diagnosis of these infections is challenging, as microorganisms become attached to the surfaces of implants, organs, bones, or tissues, and is hard to detect them circulating in the bloodstream. Therefore, their detection is difficult in samples such as blood cultures, drainage from the infection site, or synovial fluid samples, which are considered to be less invasive (Esteban et al., 2008; Drago and De Vecchi, 2017). Administration of antibiotics also reduces sensitivity in isolating the etiological microorganisms of the infection (Xu et al., 2017).

In recent years, several studies have been conducted to find diagnostic methods that offer a faster response and higher sensitivity, allowing effective treatments for these patients. One prominent technique in the study of IAIs is the sonication of the implant (Costerton et al., 2011; Drago and De Vecchi, 2017; Xu et al., 2017).

The sonication technique uses low-frequency ultrasound to detach bacterial cells from the biofilm, improving bacterial growth before culture (Apostolakis, 2020; Oliva et al., 2021). The principle of sonication is based on acoustic cavitation. When sound waves propagate in a liquid medium, they create areas of low and high pressure. In low-pressure zones, microscopic bubbles form, which collapse in high-pressure zones, releasing a large amount of energy (Joyce et al., 2003). When an implant is immersed in a liquid solution and subjected to sonication, the agitation caused by the sound waves and the energy released on its surface causes a vacuum cleaning-like action, making it possible to release adhered bacterial cells (Oliva et al., 2016).

In Clinical Microbiology, sonication is applied mostly in the study of Prosthetic Joint Infections (PJIs) (Esteban et al., 2008; Peng et al., 2023). However, there are several studies describing its application to other devices, namely CIED.

Several meta-analyses assessing the performance of sonication have been published (Apostolakis, 2020; Tsikopoulos et al., 2022; Peng et al., 2023). One meta-analysis study aimed to evaluate the accuracy of sonication in studying PJIs (including hip, knee, shoulder, and elbow joint prostheses) (Peng et al., 2023). However, neither of them has evaluated the performance of the sonication technique in the diagnosis CIED infections. Thus, this work aims to perform a systematic review and meta-analysis on this subject, to provide information that might be useful to establish future criteria for the diagnosis and treatment of these infections.

## 2 Methods

This review adhered to the Preferred Reporting Items for Systematic Reviews and Meta-Analyses (PRISMA) criteria. The proposed methodology for the systematic review was registered in the International Prospective Register of Systematic Reviews PROSPERO [CRD42023444537].

### 2.1 Search strategy and eligibility criteria

An electronic search was conducted to identify all relevant studies. The literature search was performed in MEDLINE (via PubMed) and Web of Science. The last search date was July 2023. The detailed search strategy is available in the register on PROSPERO. Only studies written in English, Spanish, and Portuguese were included to avoid any bias related to a mistranslation.

The included studies verified the following inclusion criteria:

- Studies describing the results of sonication by the analysis of CIED implants (including *Pacemakers*, ICDs, CRTs, and ILRs), removed for any reason;
- Reports that had sufficient information to compute the number of true positives (TP), true negatives (TN), false positives (FP) and false negatives (FN);
- Reports that describe the procedures adopted in the microbiology laboratory, including the sonication protocol used; incubation conditions; methods used to identify the microorganisms, and criteria used to interpret the results (only studies using traditional methods for culture (plate) and identification of microorganisms were included);
- Articles published in English, Spanish, and Portuguese.

Studies that verified any of the following criteria were excluded:

- Animal studies;
- Case studies;
- Review articles, books, opinion articles;
- *In vitro* studies;
- Results obtained by molecular methods (e.g., PCR).

### 2.2 Interventions

The intervention of interest was the sonication of the CIED implant, removed surgically from patients with CIEDIs or patients who got their device removed for reasons other than infection. The sonication was performed to detect infection or colonization of the device, including the generator and leads.

### 2.3 Outcome measures

The outcome measures of interest were sensitivity and specificity of sonication for the diagnosis of CIED infection. The results of the sterile controls were not used to calculate the overall outcome measures, however, its effect on the overall sensitivity and specificity was assessed. Additional outcomes of interest were collected for subgroup analysis.

### 2.4 Data collection

Two authors of this review independently assessed the study eligibility by inspecting the title and abstract. All articles selected from the title/abstract reading were inspected for inclusion with a full-text review by both authors. The information of all selected papers was independently extracted to a form that included study design, participants, sample size, description of intervention, outcomes, and quality assessment indicators. Discrepancies in study selection were resolved through consensus.

### 2.5 Quality assessment

Two authors independently assessed the risk of bias and the applicability of the included studies according to the QUADAS-2 tool (Whiting et al., 2011). All inconsistencies were resolved by consensus. The QUADAS-2 tool comprises several questions regarding 4 domains: Patient Selection, Index Test, Reference Standard, and Patient Flow and Timing. Applicability was evaluated for the first three domains. The overall risk of bias for each study was given by the highest score in any singular domain. When a study was at a high risk of bias in two or more domains, it was classified as being at a very high risk of bias.

A sensitivity analysis was produced to control the effect of the sample size and risk of bias in the included studies.

### 2.6 Statistical methods

After the systematic review, a meta-analysis was performed using R software (R Core Team, 2021). When the true positives (TP), true negatives (TN), false positives (FP), or false negatives (FN) were zero, the value of 0.5 was added.

Forest plots were obtained to present a graphical overview of the outcome measures. The associated confidence intervals (CIs) for the sensitivity and specificity are based on the arcsine transformation, which is a variance stabilizing transformation. Thus, the sine transformation was applied to the pooled results to re-express them as sensitivities or specificities. All estimates were computed under a random model setting obtained using the restricted maximum likelihood (REML) methodology. Between-study heterogeneity $\tau^{2}$ and the $I^{2}$ statistic was obtained via REML (Viechtbauer, 2010). All of these procedures were performed in the R package metafor (Viechtbauer, 2010). CIs were computed using the Knapp and Hartung adjustment (Hartung et al., 2008), due to the low sample size. Subgroup analysis was performed when at least 3 studies were available through a mixed-effects model.

Funnel plots, the trim and fill method (Duval and Tweedie, 2000), and Egger’s test were applied to screen for the publication bias (Hartung et al., 2008).

To jointly model the sensitivity and the false positive rate, a bivariate model which is equivalent to the hierarchical summary receiver operator characteristic model was fitted. The model was obtained using the R package mada (Doebler et al., 2010). Estimation was performed through the REML methodology with the Knapp and Hartung adjustment. The $I^{2}$ statistic of this model was computed using the Zhou and Dendukuri approach (Zhou and Dendukuri, 2014).

Two-tailed *p-*value <0.05 was considered statistically significant.

## 3 Results

### 3.1 Study selection

**Figure 1:**
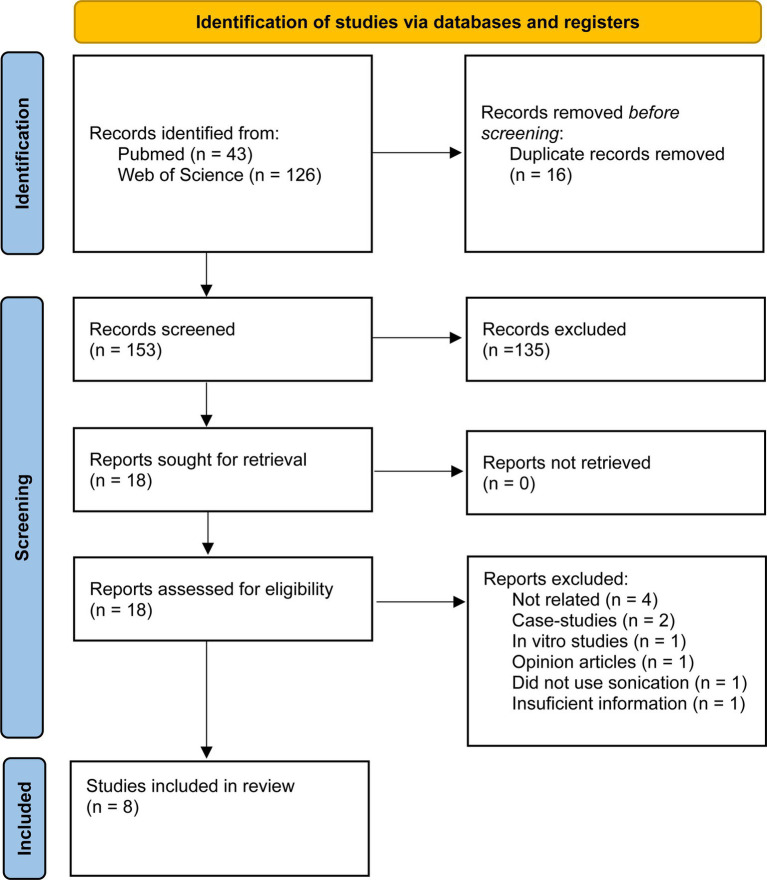
PRISMA study search flow diagram.

Through the searches in PubMed and Web of Science databases, we identified 169 records. The queries used are in Supplementary Table S1. All records were downloaded to Mendeley Reference Manager. Using this tool 16 duplicates were removed. The article screening process was performed by two authors, independently. The screening of titles and abstracts of 135 records were excluded. A full-text screening was conducted on 18 articles, resulting in the exclusion of 10 articles. Thus, the systematic literature review and meta-analysis covered 8 articles. The selection process is detailed in the PRISMA flow diagram (see Figure 1).

### 3.2 Characterization of the included studies

The main characteristics of the 8 included studies can be found in Table 1. The studies comprised 535 patients. The sample size ranged from 17 to 121. Patients were identified as patients with Pocket Infection (PI), patients with Infective Endocarditis (IE), and patients without signs of infection (NI), according to the EHRA 2019 consensus document. In Rohacek et al.’s (2014) study, 2 pacemakers from the same patient were analyzed, and removed at different times due to reinfection, leading to the study of 17 devices in 16 patients (Rohacek et al., 2014).

**Table 1:** Description of the included studies.

Author	Year	Country	Patients studied			Type of implants				T.S	Sonication protocol				
			PI	EI	NI	PPMs	ICDs	CRTs	ILRs		Vortex	F.S	D.S	C.C	Q.I.
Olsen et al. (2022)	2022	Denmark	50	60	-	68	21	21	-	NaCI	30s + 30s	S	1 min		0.2 mL
El-Ashry et al. (2021)	2021	Egypt	12	7	33	48	4	-	-	NaCI	30s + 30s	40 kHz	1 min		0.1 mL
Oliva et al. (2018)	2018	Italy	29	2	-	28	3	-	-	NaCI	30s + 30s	40 kHz	5 min	3,200 rpm, 15 min	
Inacio et al. (2015)	2015	Brazil	11	4^1^	68	72	11	-	-	R.S.	30s + 30s	40 kHz	5 min	2,500 rpm, 5 min	0.1 mL
Rohacek et al. (2014)	2014	Switzerland	14	2^2^	-	14	2	1	-	NaCI	30s + 30s	40 kHz		3,200 rpm, 20 min	0.1 mL
Oliva et al. (2013)	2013	Italy	18	2	20	34	4	-	2		30s + 30s	> 20 kHz	5 min		0.5 mL
Mason et al. (2010)	2010	USA	16	-	66	46	36	-	-	NaCI		40 kHz	5 min		
Rohacek et al. (2010)	2010	Switzerland	4	2	115	68	53	-	-	NaCI	30s + 30s	40 kHz	1 min		0.1 mL

PI, Pocket Infection; EI, Infective Endocarditis; NI, No signs of infection; 1–2 of these patients also presented PI; 2 – These patients also presented PI; PPMs, Pacemakers; ICDs, Implantable Cardioverter-Defibrillators; CRTSs, Cardiac Resynchronization Therapy devices; ILRs, Implantable Loop Recorders; − Not described in the study; T. S., Transport Solution; Vortex, Duration of the vortex step; D.S., Duration of the sonication step; C.C., Duration of the centrifugation step; Q.I, Quantity of sonicated fluid inoculated.

### 3.3 Conditions for sample collection and transport

All the studied devices were surgically removed and placed in sterilized, airtight containers and covered in a solution of NaCl at 0.9% or Ringer’s solution, to prevent drying, maintaining aseptic conditions.

Only two studies (Inacio et al., 2015; El-Ashry et al., 2021) indicated that samples were sonicated within a maximum of 6 h after removal (Inacio et al., 2015; El-Ashry et al., 2021). The remaining studies only mentioned the time of transportation to the laboratory (between 3 and 24 h), except for Olsen et al. (2022), in this study samples were stored at 5°C and processed within 48 to 72 h (Olsen et al., 2022).

### 3.4 Sonication protocol

The containers with the removed devices were processed using the VSV method (Vortex-Sonication-Vortex). In this method, there are steps of vortex agitation before and after sonication. Mason et al. (2010), did not mention vortex agitation in their procedure. Some studies added an extra step, and centrifugated the sonicated fluid to concentrate it, inoculating only the sediment obtained from centrifugation (Mason et al., 2010). The duration of the sonication step ranged between 1 and 5 min, yet, one of the studies did not describe the duration of this step (Rohacek et al., 2014). All protocols used a sound wave frequency of 40 kHz, except for the study of Oliva et al. (2013) which described a frequency > 20 kHz.

Two studies did not describe the quantity of sonication fluid inoculated in the culture media (Mason et al., 2010; Oliva et al., 2018). In the remaining studies, the inoculated quantity ranged between 0.1 mL and 0.5 mL.

All studies described the inoculation of sonicated fluid into solid culture media, incubated aerobically and anaerobically. Only some authors specified the temperature and incubation time used (Rohacek et al., 2010; Oliva et al., 2013; Rohacek et al., 2014; Inacio et al., 2015; Oliva et al., 2018).

Half of the studies further described inoculation of the sonicated fluid for 10–14 days in Thioglycolate broth (Rohacek et al., 2010, 2014; Inacio et al., 2015; Olsen et al., 2022).

The frequencies of the identified microorganisms in each study can be found in Table 2. We can observe that coagulase-negative *Staphylococcus* spp. (CNS) and *Staphylococcus aureus* were the most isolated microorganisms.

**Table 2:** Frequencies of the identified microorganisms on each study.

Author, Publication Year	CNS		*S. aureus*	*C. acnes*	*GNB*				*Corynebacterium* spp.	*Enterococcus* spp.	*Streptococcus* spp.	Other Microorganisms ^4^	No Growth
	*S. epidermidis*	Other species ^2^			*P. aeruginosa*	*Klebsiella* spp.	*A. baumannii*	Other species^3^					
Olsen et al. (2022) ^1^	22.73%	5.45%	28.18%	8.18%	4.54%				5.45%	9.09%	8.18%	2.72%	5.45%
El-Ashry et al. (2021)	28.85%		9.62%	0%	1.92%	7.69%^5^	0%	5.77%	0%	0%	1.92%	1.92%	42.31%
Oliva et al. (2018)	65.53%	11.91%	3.97%	0%	5.29%		0%		5.29%	0%	0%	0%	10%
Inacio et al. (2015)	26.51%		7.23%	0%	2.41%	3.62%	3.62%	9.64%	1.2%	0%	10.84%	4.81%	30.12%
Rohacek et al. (2014)	25%		50%^6^	12.5%^7^	0%				0%	0%	0%	6.25%	6.25%
Oliva et al. (2013)	40%	15%	2.5%	0%	3.75%	1.25%	0%	1.25%	2.5%	0%	0%	1.25%	32.5%
Mason et al. (2010)	3.65%	6.1%	6.1%	4.88%	8.53%				0%	0%	0%	2.44%	68.29%
Rohacek et al. (2010)	12.4%		0.82%	22.31%	0.82%				0%	0%	0.82%	4.13%^8^	58.68%

CNS, Coagulase negative Staphylococcus; GNB, Gram negative Bacilli; ^1^Microorganisms identified in tissue cultures, swab cultures, hemocultures and sonicated fluid cultures, (by MALDI-TOF MS or NGS); ^2^*S. homins, S. hemoliticus, S. capitis, S. lugdunensis*; ^3^*Escherichia coli, Serratia marcescens, Proteus mirabilis, Stenotrophomonas maltophilia, Enterobacter spp., Chryseobacterium indologenes, Brevundimonas spp., Serratia marcescens*; ^4^*Kytococcus schroeteri, Gordonia terrae, Candida albicans, Micrococus* spp.*, Bacillus cereus, Peptostreptococcus* spp.*, Finegoldia* spp.; ^5^One culture (1.92%) showed polymicrobial growth with *Proteus mirabilis*; ^6^Two cultures (12.5%) showed polymicrobial growth with *P. acnes* and *P. aeruginosa*; ^7^One culture (6.25%) showed polymicrobial growth with CNS; ^8^One culture (0.82%) showed polymicrobial growth, however the species where not described by the authors.

### 3.5 Performance of sonication

**Figure 2:**
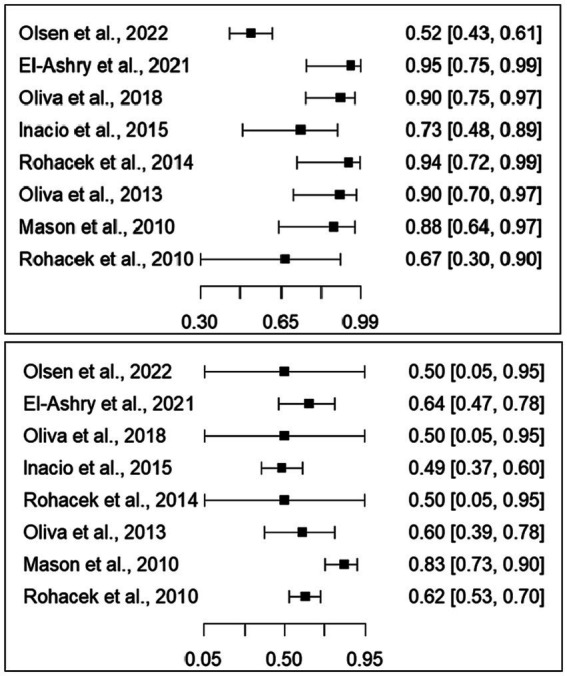
Forest plots for sensitivity (top) and specificity (bottom). The squares represent the values of sensitivity and specificity, and the lines represent the confidence intervals at 95%.

Sensitivity values ranged from 0.52 (95% CI: 0.43–0.61) to 0.95 (95% CI: 0.75–0.99). Specificity values ranged from 0.49 (95% CI: 0.37–0.60) to 0.83 (95% CI: 0.73–0.90). Details can be found in the forest plots (Figure 2).

**Figure 3:**
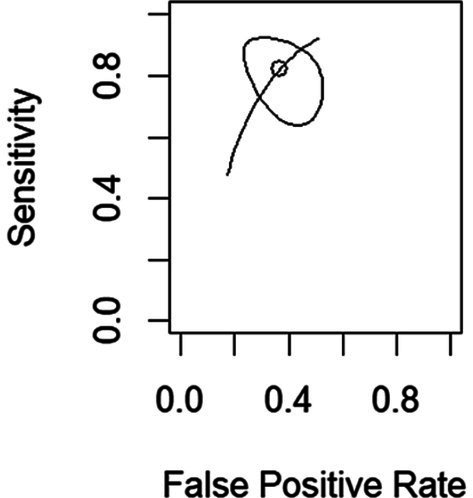
SROC curve for the sonication method. The circle represents the overall estimate, and the ellipsis represents the 95% confidence region.

A Summary Receiver Operating Characteristic Curve (SROC) was plotted for the bivariate model (see Figure 3). The partial area under the SROC curve for the sonication method is 0.754.

The overall sensitivity and specificity of the sonication method was 0.823 (95% CI: 0.682–0.910), with an *I*^2^ of 14.3%. The overall specificity was 0.632 (95% CI: 0.506–0.743).

### 3.6 Subgroup analysis

A subgroup analysis was performed to identify which factors can influence sensitivity and/or specificity. Hence, a mixed effects model was fitted for each of the following factors: Ethnicity; Diabetes Mellitus; Hearth failure; Chronic renal failure; History of infection; History of previous surgeries; Mean age; Antibiotic use before removal; Centrifugation; Quantity inoculated; Transport solution. The results are displayed in Table 3.

**Table 3:** Subgroup analysis according to several factors (95% confidence intervals are presented in brackets).

		No. of studies	Sensitivity (95% CI)	Specificity (95% CI)	I^2^ statistic^a^	p^b^
Ethnicity	Europe or North America	5	76.6 (69.4–82.9)	57.5 (55.5–59.5)	43.2/5.1	0.835/0.179
	Other	2	76.1 (0.0–81.5)	52.2 (0.0–100.0)		
Diabetes Mellitus	≤50%	4	76.0 (63.8–86.0)	60.0 (37.1–78.7)	27.5/87.6	0.436/0.972
	>50%	1	81.2 (74.9–86.6)	59.4 (45.5–71.8)		
Hearth failure	≤50%	2	76.1 (0.0–100.0)	57.8 (46.4–68.2)	35.4/82.5	0.892/0.779
	>50%	3	77.5 (60.2–90.3)	60.9 (21.1–89.0)		
Chronic renal failure	>15%	4	76.0 (63.8–86.0)	60.0 (37.1–78.7)	27.5/87.6	0.436/0.972
	≤15%	1	81.2 (74.9–86.6)	59.4 (45.5–71.8)		
History of infection	3%	2	65.5 (33.7–88.6)	52.9 (0.0–98.4)	0.0/49.0	0.01*/0.525
	6%	2	81.0 (78.7–83.3)	59.1 (34.3–79.3)		
History of previous surgeries	≤20%	2	65.5 (33.7–88.6)	52.9 (0.0–98.4)	28.6/30.5	0.175/0.752
	>20%	4	77.1 (68.8–84.2)	55.4 (50.2–60.4)		
Mean age	≤70%	4	79.2 (69.7–87.1)	54.5 (44.0–64.2)	0.0/50.0	0.736/0.081
	>70%	3	78.1 (76.0–80.1)	66.6 (35.0–89.1)		
Antibiotic use before removal	≤70%	4	79.4 (72.7–85.3)	52.7 (41.5–63.1)	5.6/57.1	0.211/0.096
	>70%	4	74.4 (64.9–82.6)	65.2 (47.2–80.0)		
Centrifugation	Centrifugation	4	78.4 (65.9–88.4)	46.7 (46.0–47.3)	32.4/55.7	0.697/0.064
	No centrifugation	4	76.2 (68.4–82.9)	63.0 (51.6–73.3)		
Quantity inoculated	0.1 mL	4	73.8 (0.0–99.4)	79.2 (69.7–87.1)	40.4/27.4	0.501/0.856
	>0,1 mL	2	79.2 (69.7–87.1)	73.8 (0.0–99.4)		
Transport solution	0.9% NaCl	6	77.4 (71.7–82.6)	63.7 (53.7–72.7)	33.3/56.0	0.656/0.089
	Other	2	74.6 (0.0–96.1)	49.0 (0–90.4)		

A *p*-value lower than 0.05 is identified with *; a: *I*^2^ statistic is presented for the sensitivity and specificity of the respective mixed effect models; test for the significance of each factor in the mixed models for sensitivity and specificity.

We observed substantial heterogeneity in some situations when considering sensitivity and specificity in separate models. For the specificity models the *I*^2^ is higher than 80%.

For the sensitivity mixed effects models, only the history of infection showed a significant effect with sensitivity increasing about 15% when the history of infection doubles from 3 to 6% (*p* = 0.01).

For the specificity mixed effects models, no significant factor was found.

### 3.7 Risk of bias assessment

The included studies were scored by the Quality Assessment of Diagnosis Accuracy Studies (QUADAS 2) tool (Whiting et al., 2011) to assess the risk of bias and the applicability of the diagnostic studies.

As for the Index Test, all studies were rated as having a high risk of bias because it was not performed blindly. The sonication process was conducted with the knowledge of the patient’s infection characteristics.

In the Index Test domain, all articles were considered as at a high risk of bias. Thus, evaluating the QUADAS-2 domains for risk of bias and applicability, three of the included articles were also considered high-risk studies in other domains, therefore we considered them as being at a very high risk of bias.

To assess the impact of high-risk studies, we removed studies considered to have a higher risk based on the QUADAS-2 analysis and calculated sensitivity and specificity estimates without including these studies. As all studies were at a high risk of bias in at least one domain, the 5 studies at a high risk of bias for only one domain were compared to the other 3 studies. No significant differences were found in the pooled sensitivity (*p* = 0.504) and pooled specificity (*p* = 0.641).

### 3.8 Publication bias assessment

Funnel plots for the random models for sensitivity and specificity were generated for publication bias assessment (*cf.* Supplementary Figures S1A,B). Egger’s test *p*-values were 0.582 and 0.543 for the sensitivity and specificity models. The trimm and fill method did not find any missing study in either model. Thus, the results suggest that publication bias was not present.

## 4 Discussion

The lack of standard diagnostic methods and well-defined infection criteria is a major difficulty in diagnosing IAIs (VanEpps and Younger, 2016).

The formation of biofilm and the prevalence of low virulent microorganisms in these types of infections are also a difficulty (Oliva et al., 2021). Sonication emerges as a response to improve the detection of these pathogens (Spindler et al., 2022). We found that Gram-positive cocci were the most identified species across studies. *S. aureus* was predominant in three studies, while CNS, particularly *S. epidermidis*, was frequent in four studies. Other species like *Staphylococcus hominis, Staphylococcus haemolyticus*, and *Staphylococcus capitis* were also noted. These species are widely recognized as biofilm producers. Also, in Rohacek et al. (2010), *Cutibacterium acnes* was the most common, followed by CNS. *C. acnes* is also recognized as fastidious microorganism, that can cause latent infections without manifestations.

In the present study, patients were divided into three groups (patients with IBS, patients with IE, and patients without signs of infection) according to these criteria. However, none of the 8 studies included in this review reference the EHRA 2019, and 6 of these articles were published before the consensus document.

The patients in these studies were considered as having IE using the modified Duke criteria (Li et al., 2000), and as having PI, through the presence of characteristics that were found to be well described in the studies, thus allowing these patients to be covered in the EHRA 2019 criteria (Bongiorni et al., 2018). Only one study by Oliva et al. (2018), did not describe the manifestations of patients with PI and only referenced the Duke criteria (Oliva et al., 2018).

In the present study, sonication demonstrated a sensitivity of 0.823 (95% CI: 0.682–0.910) and specificity of 0.632 (95% CI: 0.506–0.743) in the diagnosis of infections associated with CIED. Due to this low specificity, sonication only shows an acceptable performance (PAUC is 0.754) as a diagnostic method. The ability to identify infected individuals is good, however, the specificity is not, corroborating the recommendations of the EHRA 2019, which states that the results obtained through sonication must be carefully interpreted. These results are also in agreement with previous studies on the use of sonication in the field of orthopedics and neurosurgery (Apostolakis, 2020; Peng et al., 2023). In the study by Peng et al. (2023), regarding the diagnosis of OI, a high value for specificity was obtained: 0.96 (95% CI: 0.95–0.96) (Peng et al., 2023). This can be explained by the definition of lower cut-off values in the diagnosis of these infections. In the present study, cut-off values were not evaluated, and culture results were interpreted qualitatively. Still, in the diagnosis of PJIs, a high level of heterogeneity between studies has been described, due to some inconsistent results (Peng et al., 2023). In the study by Apostolakis (2020), the specificity of sonication in diagnosing external ventricular shunt drain infections was 0.571 (95% CI: 0.230–0.856), leading the authors to consider that this method should not be applied alone in the evaluation of these infections.

The sensitivity calculated for this method, for which the value was 0.823 (95% CI: 0.682–0.910), demonstrates that its use offers advantages in the diagnosis of infections associated with CIED.

By performing a subgroup analysis, we observed that only the history of infection had an impact on the sensitivity values. A higher rate of patients with a history of infection resulted in a 15% higher sensitivity. In implant-associated infections, a history of previous infection leads to a higher risk of reinfection (Oliva et al., 2021) which is probably associated with a higher prevalence of infection in these patients. The role of the prevalence rate has already been discussed in several other contexts (Kurtz et al., 2012). These results are not aligned with findings from other studies on the question of previous antimicrobial therapy. In studies about sonication of hip and knee prostheses, the use of antibiotics before the removal of the prosthesis reduced the sensitivity of the sonicated fluid cultures (Trampuz et al., 2007).

For specificity, no factor had a significant effect. However, age, antibiotic use, concentration by centrifugation, and transport solution would have an impact on specificity at a 10% significance level. Some authors advise researchers to use a higher significance level when the number of studies is low to balance the test power with the significance level (Hartung et al., 2008). Thus, specificity may increase with the age of the individuals (*p* = 0.081) and with the use of antibiotics (*p* = 0.096). Not using centrifugation (*p* = 0.064) and using a 0.9% NaCl transport solution (*p* = 0.089) may be associated with a higher specificity. However, these conclusions need further investigation as they rely on a low number of studies.

Previous studies showed that the use of sonication together with a large spectrum of culture media increases the sensitivity of the diagnosis, due to the prevalence of low virulent pathogens in IAIs (Esteban et al., 2008). In the present study, we found insufficient information for a subgroup analysis on the culture media used. This variable needs to be assessed in future studies. The combination of sonication with other methods of microbial detection and identification also needs more research. Multiplex-PCR has been useful in the diagnosis of PJIs in samples such as synovial fluid or blood cultures, and some studies already described its application to sonicated fluid. Yet, applying these techniques to IAIs still needs more research since they can lead to false-positive results (Esteban et al., 2008; Corvec et al., 2012).

This review’s limitation is the number of studies included. Although, the results are “robust” as the sensitivity analysis did not show a relation between the quality of the studies and the results. Moreover, no evidence of publication bias was found. Our findings suggest that sonication may offer advantages in diagnosing these infections.

Further studies are needed to improve diagnostic methods and reach a consensus on the standard laboratory algorithm for this type of infection, as well as a better understanding of the characteristics of infections associated with CIED, leading to a more accurate diagnosis and effective treatment of these conditions. In this context, this work provides some insight into the performance of sonication, which is a method that could be useful to define this algorithm.

In 2023 the ESC created new guidelines for the management of Endocarditis. This document provides some new insights into the management of CIED, such as recommendations related to pre-procedural aseptic measures of the site of implantation, to the device extraction if there is a definite diagnosis of CIED-related IE, and to the management of antimicrobial therapy (Delgado et al., 2023). These new ESC guidelines were published after the present study was conducted; therefore, we followed the EHRA 2019 guidelines when including patients with IE. In future studies, authors should consider these new guidelines, as they offer standardized and updated procedures for these scenarios.

## Data availability statement

The original contributions presented in the study are included in the article/Supplementary material, further inquiries can be directed to the corresponding author.

## Author contributions

DA: Investigation, Methodology, Supervision, Writing – original draft, Writing – review & editing. JM: Methodology, Software, Supervision, Writing – original draft, Writing – review & editing. SF: Methodology, Supervision, Validation, Writing – original draft, Writing – review & editing. SM: Investigation, Methodology, Supervision, Validation, Writing – original draft, Writing – review & editing. PF: Formal analysis, Writing – review & editing. RP: Formal analysis, Writing – review & editing.

## References

[ref1] Apostolakis S. (2020). Use of focused ultrasound (sonication) for the diagnosis of infections in neurosurgical operations: A systematic review and Meta-analysis. World Neurosurg. 136, 364–373.e2. doi: 10.1016/j.wneu.2019.12.143 31899387

[ref2] Blomström-Lundqvist C. Traykov V. Erba P. A. Nielsen H. B. J. C. Bongiorni M. G. (2020). European heart rhythm association (EHRA) international consensus document on how to prevent, diagnose, and treat cardiac implantable electronic device infections-endorsed of clinical microbiology and infectious diseases (ESCMID) in collaboration with the European Association for Cardio-Thoracic Surgery (EACTS). Europace 22, 515–549. doi: 10.1093/europace/euz246 31702000 PMC7132545

[ref3] Bongiorni M. G. Burri H. Deharo J. C. Starck C. Kennergren C. Saghy L. . (2018). 2018 EHRA expert consensus statement on Lead extraction: Recommendations on definitions, endpoints, research trial design, and data collection requirements for clinical scientific studies and registries: Endorsed by APHRS/HRS/LAHRS. Europace 20:1217. doi: 10.1093/europace/euy050 29566158

[ref4] Corvec S. Portillo M. E. Pasticci B. M. Borens O. Trampuz A. (2012). Epidemiology and new developments in the diagnosis of prosthetic joint infection. Int. J. Art. Organs 35, 923–934. doi: 10.5301/ijao.5000168, PMID: 23138706

[ref5] Costerton J. W. Post J. C. Ehrlich G. D. Hu F. Z. Kreft R. Nistico L. . (2011). New methods for the detection of orthopedic and other biofilm infections. FEMS Immunol. Med. Microbiol. 61, 133–140. doi: 10.1111/j.1574-695X.2010.00766.x 21204998

[ref6] Darouiche R. O. (2001). Device-associated infections: a macroproblem that starts with microadherence. Clin. Infect. Dis. 33:1572. doi: 10.1086/323130, PMID: 11577378

[ref7] Delgado V. Ajmone Marsan N. de Waha S. Bonaros N. Brida M. Burri H. . (2023). 2023 ESC guidelines for the Management of Endocarditis. Eur. Heart J. 44, 3948–4042. doi: 10.1093/eurheartj/ehad193, PMID: 37622656

[ref8] Doebler P. Holling H. Sousa-Pinto B. (2010). Meta-analysis of diagnostic accuracy with Mada. Available at: http://r-forge.r-project.org/projects/mada/

[ref9] Drago L. De Vecchi E. (2017). Microbiological diagnosis of implant-related infections: scientific evidence and cost/benefit analysis of routine Antibiofilm processing. Adv. Exp. Med. Biol. 971, 51–67. doi: 10.1007/5584_2016_154, PMID: 27815925

[ref10] Duval S. Tweedie R. (2000). Trim and fill: a simple funnel-plot-based method of testing and adjusting for publication bias in meta-analysis. Biometrics 56, 455–463. doi: 10.1111/j.0006-341X.2000.00455.x, PMID: 10877304

[ref11] El-Ashry A. H. Mohammed S. A. Hussein K. S. El Elhoufey A. (2021). Clinical utility of sonication for diagnosing infection and colonization of cardiovascular implantable electronic devices. Med. Microbiol. Immunol. 210, 245–250. doi: 10.1007/s00430-021-00717-2, PMID: 34254192

[ref12] Esteban J. Gomez-Barrena E. Cordero J. Martín-de-Hijas N. Z. Kinnari T. J. Fernandez-Roblas R. (2008). Evaluation of quantitative analysis of cultures from sonicated retrieved orthopedic implants in diagnosis of orthopedic infection. J. Clin. Microbiol. 46, 488–492. doi: 10.1128/JCM.01762-07, PMID: 18077647 PMC2238112

[ref13] Haghjoo M. (2017). “Cardiac Implantable Electronic Devices” in Practical cardiology. eds. Maleki M. Alizadehasl A. Haghjoo M. (St. Louis, MI: Elsevier), 251–260.

[ref14] Hartung J. Knapp G. Sinha B. (2008). Statistical meta-analysis with applications. Hoboken, NJ: Wiley.

[ref15] Inacio R. C. Klautau G. B. Maria A. S. Murça C. B. Da Silva S. N. Rivetti L. A. . (2015). Microbial diagnosis of infection and colonization of cardiac implantable electronic devices by use of sonication. Int. J. Infect. Dis. 38, 54–59. doi: 10.1016/j.ijid.2015.07.018, PMID: 26216762

[ref16] Joyce E. Phull S. S. Lorimer J. P. Mason T. J. (2003). The development and evaluation of ultrasound for the treatment of bacterial suspensions. A study of frequency, power and sonication time on cultured Bacillus species. Ultrason. Sonochem. 10, 315–318. doi: 10.1016/S1350-4177(03)00101-9, PMID: 12927605

[ref17] Kamath A. F. Ong K. L. Lau E. Chan V. Vail T. P. Rubash H. E. . (2015). Quantifying the burden of revision Total joint arthroplasty for Periprosthetic infection. J. Arthroplasty 30, 1492–1497. doi: 10.1016/j.arth.2015.03.035, PMID: 25865815

[ref18] Kurtz S. M. Lau E. Watson H. Schmier J. K. Parvizi J. (2012). Economic burden of periprosthetic joint infection in the United States. J. Arthroplasty 27, 61–65.e1. doi: 10.1016/j.arth.2012.02.022, PMID: 22554729

[ref19] Li J. S. Sexton D. J. Mick N. Nettles R. Fowler V. G. Ryan T. . (2000). Proposed modifications to the Duke criteria for the diagnosis of infective endocarditis. Available at: http://cid.oxfordjournals.org/10.1086/313753 10770721

[ref20] Mason P. K. Dimarco J. P. Ferguson J. D. Srijoy Mahapatra J. Mangrum M. Bilchick K. C. . (2010). Sonication of explanted cardiac rhythm management devices for the diagnosis of pocket infections and asymptomatic bacterial colonization. Pacing Clin. Electrophysiol. 34, 143–149. doi: 10.1111/j.1540-8159.2010.02820.x, PMID: 20561226 PMC4555211

[ref21] Oliva A. Mascellino M. Nguyen B. De Angelis M. Cipolla A. Di Berardino A. . (2018). Detection of biofilm-associated implant pathogens in cardiac device infections: high sensitivity of sonication fluid culture even in the presence of antimicrobials. J. Global Infect. Dis. 10, 74–79. doi: 10.4103/jgid.jgid_31_17, PMID: 29910567 PMC5987375

[ref22] Oliva A. Miele M. al Ismail D. di Timoteo F. de Angelis M. Rosa L. . (2021). Challenges in the microbiological diagnosis of implant-associated infections: a summary of the current knowledge. Front. Microbiol. 12:750460. doi: 10.3389/fmicb.2021.750460, PMID: 34777301 PMC8586543

[ref23] Oliva A. Nguyen B. L. Mascellino M. T. D’Abramo A. Iannetta M. Ciccaglioni A. . (2013). Sonication of explanted cardiac implants improves microbial detection in cardiac device infections. J. Clin. Microbiol. 51, 496–502. doi: 10.1128/JCM.02230-12, PMID: 23196364 PMC3553873

[ref24] Oliva A. Pavone P. D’Abramo A. Iannetta M. Mastroianni C. M. Vullo V. (2016). Role of sonication in the microbiological diagnosis of implant-associated infections: beyond the orthopedic prosthesis. Adv. Exp. Med. Biol. 897, 85–102. doi: 10.1007/5584_2015_5007, PMID: 26566645

[ref25] Olsen T. Justesen U. S. Nielsen J. C. Jørgensen O. D. Sandgaard N. C. F. Ravn C. . (2022). Microbiological diagnosis in cardiac implantable electronic device infections detected by sonication and next-generation sequencing. Heart Rhythm. 19, 901–908. doi: 10.1016/j.hrthm.2022.01.039, PMID: 35124230

[ref26] Peng G. Liu Q. Guan Z. Liu M. Sun X. Zhu X. . (2023). Diagnostic accuracy of sonication fluid cultures from prosthetic components in Periprosthetic joint infection: an updated diagnostic meta-analysis. J. Orthop. Surg. Res. 18:175. doi: 10.1186/s13018-023-03662-3, PMID: 36890571 PMC9996915

[ref1002] R Core Team (2021). R: A language and environment for statistical computing. R Foundation for Statistical Computing. Vienna, Austria.

[ref27] Rohacek M. Erne P. Kobza R. Pfyffer G. E. Frei R. Weisser M. (2014). Infection of cardiovascular implantable electronic devices: detection with sonication, swab cultures, and blood cultures. Pacing Clin. Electrophysiol. 38, 247–253. doi: 10.1111/pace.12529, PMID: 25377386

[ref28] Rohacek M. Weisser M. Kobza R. Schoenenberger A. W. Pfyffer G. E. Frei R. . (2010). Bacterial colonization and infection of electrophysiological cardiac devices detected with sonication and swab culture. Circulation 121, 1691–1697. doi: 10.1161/CIRCULATIONAHA.109.906461, PMID: 20368521

[ref29] Spindler P. Faust K. Finger T. Schneider G. H. Bayerl S. Trampuz A. . (2022). High frequency of low-virulent microorganisms detected by sonication of implanted pulse generators: so what? Stereotact. Funct. Neurosurg. 100, 8–13. doi: 10.1159/000517472, PMID: 34488223

[ref30] Trampuz A. Piper K. E. Jacobson M. J. Hanssen A. D. Unni K. K. Osmon D. R. . (2007). Sonication of removed hip and knee prostheses for diagnosis of infection. Available at: www.nejm.org 10.1056/NEJMoa061588 17699815

[ref31] Tsikopoulos K. Christofilos S. I. Kitridis D. Sidiropoulos K. Stoikos P. N. Gravalidis C. . (2022). Is sonication superior to Dithiothreitol in diagnosis of Periprosthetic joint infections? A meta-analysis. Int. Orthop. 46, 1215–1224. doi: 10.1007/s00264-022-05350-z 35199219

[ref32] VanEpps J. S. Younger J. G. (2016). Implantable device-related infection. Shock 46, 597–608. doi: 10.1097/SHK.0000000000000692, PMID: 27454373 PMC5110396

[ref33] Viechtbauer W. (2010). Conducting meta-analyses in R with the meta for package. J. Stat. Softw. 36, 1–48. doi: 10.18637/jss.v036.i03

[ref34] Whiting P. F. Rutjes A. W. S. Westwood M. E. Mallett S. Deeks J. J. Reitsma J. B. . (2011). QUADAS-2: A revised tool for the quality assessment of diagnostic accuracy studies. Available at: www.annals.org 10.7326/0003-4819-155-8-201110180-00009 22007046

[ref35] Xu Y. Larsen L. H. Lorenzen J. Hall-Stoodley L. Kikhney J. Moter A. . (2017). Microbiological diagnosis of device-related biofilm infections. APMIS 125, 289–303. doi: 10.1111/apm.12676 28407422

[ref36] Zhou Y. Dendukuri N. (2014). Statistics for quantifying heterogeneity in univariate and bivariate meta-analyses of binary data: the case of meta-analyses of diagnostic accuracy. Stat. Med. 33, 2701–2717. doi: 10.1002/sim.6115 24903142

