# Corrigendum: A meta-analysis on the role of sonication in the diagnosis of cardiac implantable electronic device-related infections

**DOI:** 10.3389/fmicb.2025.1567933

**Published:** 2025-02-11

**Authors:** Daniela Araújo, João P. Martins, Stephanie Lopes Ferreira, Sandra Mota, Pedro L. Ferreira, Rui Pimenta

**Affiliations:** ^1^Escola Superior de Saúde, Instituto Politécnico do Porto, Rua Dr. António Bernardino de Almeida, Porto, Portugal; ^2^CEAUL– Centro de Estatística e Aplicações, Faculdade de Ciências, Universidade de Lisboa, Campo Grande, Lisbon, Portugal; ^3^CHUdSA – Centro Hospitalar Universitário de Santo António, Porto, Portugal; ^4^REQUIMTE/LAQV, Escola Superior de Saúde, Instituto Politécnico do Porto, Rua Dr. António Bernardino de Almeida, Porto, Portugal; ^5^Faculty of Economics, University of Coimbra, Coimbra, Portugal; ^6^Centre for Health Studies and Research of University of Coimbra, Centre for Innovative Biomedicine and Biotechnology, Avenida Dias da Silva, Coimbra, Portugal

**Keywords:** sonication, implant associated infections, cardiac implantable electronic devices, biofilms, microbiological diagnosis, systematic review, meta-analysis

In the published article, there was an error in affiliations 3, 4, 5, and 6. Instead of “Daniela Araújo^1*^, João P. Martins^1, 2, 3^, Stephanie Lopes Ferreira^1, 4^, Sandra Mota^1, 5*^, Pedro L. Ferreira^3, 6^ and Rui Pimenta^1, 3^”, it should be “Daniela Araújo^1*^, João P. Martins^1, 2^, Stephanie Lopes Ferreira^1, 3^, Sandra Mota^1, 4*^, Pedro L. Ferreira^5, 6^ and Rui Pimenta^1, 6^.”

The authors apologize for this error and state that this does not change the scientific conclusions of the article in any way. The original article has been updated.

