# Supplementary material for: A meta-analysis on the role of sonication in the diagnosis of cardiac implantable electronic device-related infections
Source: Front Microbiol. 2024 Mar 15;15:1361626. doi: 10.3389/fmicb.2024.1361626 (PMC10978762; doi:10.3389/fmicb.2024.1361626)
Supplement: Supplementary file 1 [file Table_1.DOCX]

**Supplementary Table S1**

| Database | Query | | Results |
| --- | --- | --- | --- |
| PubMed | *("Sonication"[MeSH Terms] AND "Cardiac Surgical Procedures"[MeSH Terms]) OR ("Sonication"[MeSH Terms] AND "heart, artificial"[MeSH Terms]) OR ("Sonication"[MeSH Terms] AND "pacemaker, artificial"[MeSH Terms]) OR ("Biofilms"[MeSH Terms] AND "Cardiac Surgical Procedures"[MeSH Terms]) OR ("Biofilms"[MeSH Terms] AND "heart, artificial"[MeSH Terms]) OR ("Biofilms"[MeSH Terms] AND "pacemaker, artificial"[MeSH Terms])* | | 43 |
| Web of Science | #1 | *(ALL=(Sonication) AND ALL=(Pacemaker)) OR (ALL=(Biofilm) AND ALL=(Pacemaker))* | 82 |
|  | #2 | *(ALL=(Sonication) AND ALL=(Artificial Heart)) OR (ALL=(Biofilm) AND ALL=(Artificial Heart))* | 55 |
|  | #3 | *(ALL=(Sonication) AND ALL=(Cardiac Implantable Devices)) OR (ALL=(Biofilm) AND ALL=( Cardiac Implantable Devices))* | 45 |
|  | #4 | *(ALL=(Sonication) AND ALL=(Cardiac Electronic Devices)) OR (ALL=(Biofilm) AND ALL=(Cardiac Electronic Devices))* | 37 |
|  | #5 | *(ALL=(Sonication) AND ALL=(Cardiac Surgical Procedures)) OR (ALL=(Biofilm) AND ALL=(Cardiac Surgical Procedures))* | 5 |
|  | #6 | *(ALL=(Sonication) AND ALL=(Cardiac Resynchronization Therapy Devices)) OR (ALL=(Biofilm) AND ALL=(Cardiac Resynchronization Therapy Devices))* | 2 |
|  | #7 | *#1 OR #2 OR #3 OR #4 OR #5 OR #6 and Review Article (Exclude – Document Types)* | 126 |
