# Supplementary figures and images for: A meta-analysis on the role of sonication in the diagnosis of cardiac implantable electronic device-related infections
Source: Front Microbiol. 2024 Mar 15;15:1361626. doi: 10.3389/fmicb.2024.1361626 (PMC10978762; doi:10.3389/fmicb.2024.1361626)

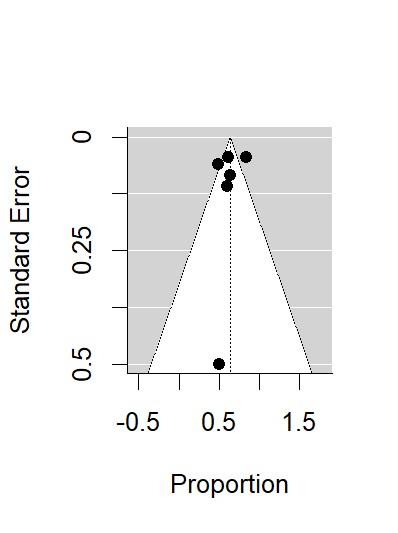

Supplement: Supplementary file 2 [file Image_1.jpeg]

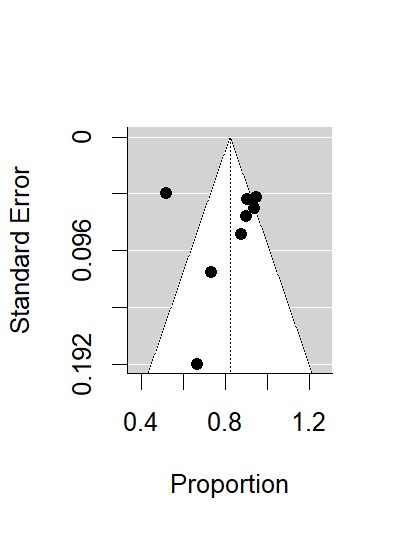

Supplement: Supplementary file 3 [file Image_2.jpeg]
